# Remission or Persistence? A Prediction Tool to Identify Women at Risk for Long-Term Depressive Symptoms Postpartum

**DOI:** 10.1155/2024/7734542

**Published:** 2024-08-30

**Authors:** Karin Gidén, Richelle Duque Björvang, Richard Aubrey White, Lisa Vinnerljung, Stavros I Iliadis, Alkistis Skalkidou, Emma Fransson

**Affiliations:** ^1^Department of Women's and Children's Health, Uppsala University 751 85, Uppsala, Sweden; ^2^Department of Method Development and Analytics, Norwegian Institute of Public Health, Oslo, Norway; ^3^Region Gävleborg, Gävleborg, Sweden; ^4^Centre for Translational Microbiome Research, Department of Microbiology, Tumor and Cell Biology, Karolinska Institutet, Stockholm 171 65, Sweden

## Abstract

**Background:**

Peripartum depression is a common complication with potential long-term adverse effects on the woman and her family. Approximately 30%–50% of newly delivered women experience prolonged depressive symptoms at 6–12 months postpartum. Early detection may facilitate preventive and treatment interventions.

**Aim:**

To investigate correlates for and create a tool for predicting long-term symptomatology in women experiencing depressive symptoms at 6 weeks postpartum.

**Materials and Methods:**

Data from the Biology, Affect, Stress, Imaging, and Cognition study was used, to identify women who scored high (≥12) on the Edinburgh Postnatal Depression Scale (EPDS) at 6 weeks postpartum (*n* = 697). Further, we collected data from medical records and included 40 variables based on earlier studies and clinical experience. A total of 654 women were included. Elastic net linear regression analysis was performed to identify predictors of continued symptoms at 6 months postpartum. An equation predicting the EPDS score at 6 months postpartum based on weighted variables was developed.

**Results:**

High education level and sleep for more than 6 hr per night in pregnancy week 17 were protective factors. Parity, pregnancy complications, stressful events, attention deficit hyperactivity disorder/attention deficit disorder, history of depression, depressive symptoms, and anxiety during pregnancy were predictive factors of prolonged depressive symptoms. A prediction tool with area under curve 0.73 and positive predictive value of 79%–83% depending on chosen EPDS cutoff was developed for clinical use.

**Conclusions:**

Our prediction tool offers a method to identify women at risk for persisting depressive symptoms postnatally, based on their significant depressive symptoms during the first weeks after delivery. Screening in order to identify these women can already start in the antenatal setting.

## 1. Introduction

Peripartum depression (PPD) is a common condition after childbirth with a global prevalence estimated around 17% [1]. Multiple risk factors for PPD have been identified, such as low education level, low socioeconomic status, smoking, multiparity, history of mental illness and especially depression, history of abuse, and level of support [2, 3, 4, 5]. For a proportion of women, PND can trigger onset of chronic mental health issues [6, 7]. Not only does PPD impact the woman experiencing it, but it also has a profound effect on her family and the community [8]. Mounting evidence shows that the infant is at increased risk for a spectrum of disturbances in physical and developmental health [9, 10, 11, 12, 13, 14, 15]. In several large longitudinal cohort studies, the severity and chronicity of maternal symptoms have been correlated to an increased risk for emotional and behavioral difficulties in preschool age in offspring [16, 17, 18, 19].

The proportion of women with persistent symptoms of depression at 12 months postpartum is estimated to be 30%–50% depending on the setting, according to a review of longitudinal studies [20]. However, long-term postpartum depressive symptoms (LTPDS) are less explored and the few published results seem to be inconsistent. Smaller studies have reported various LTPDS-associated factors: low income, multiparity, low social support, stressful events, severe symptoms early postpartum, and depressive symptoms during pregnancy [21, 22, 23]. Parity, education, baseline global functioning, and depression severity have been reported as factors that can distinguish chronic severe trajectory from gradual remission and partial improvement of symptoms [24]. A clinical tool to differentiate between short- and long-term depressive symptoms provides a possibility to make evidence-based decisions easier and faster for healthcare personnel. Today, no such tool is available for use in clinical practice.

Recently, there has been a growing emphasis on examining various trajectories of PND that exhibit distinct characteristics [25]. This is usually achieved by monitoring depressive symptoms over a period of a couple of years. Previous studies have attempted to assess depressive symptoms long-term, by retrospectively categorizing women into groups based on the severity and progression of depressive symptoms. However, in a review of 11 trajectory studies, Baron et al. [26] reported an inconsistency in predicting factors for the different trajectories across studies. Nevertheless, trajectory modeling typically does not involve clinical tools that can accurately predict which individuals are at a higher risk of developing more severe symptoms.

The aim of this study was therefore to identify factors predicting LTPDS by 6 months postpartum, in a population of newly delivered mothers reporting depressive symptoms at 6 weeks postpartum and to create a clinically easy-to-use prediction tool to identify women at risk of LTPDS. This approach exploring easily recognizable and clinically relevant predictive factors for LTPDS in this group of women is novel and has, to our knowledge, not been pursued before.

## 2. Materials and Methods

### 2.1. Participants

The Biology, Affect, Stress, Imaging, and Cognition (BASIC) project was undertaken at the Department of Obstetrics and Gynecology at Uppsala University Hospital, Sweden. The BASIC project was a prospective, longitudinal population-based cohort study, longitudinally following 6,478 pregnancies from 2009 to 2018 [27]. The project included web questionnaires with the Edinburgh Postnatal Depression Scale (EPDS) in pregnancy weeks 17 and 32 and postpartum at 6 weeks and 6 months. Questions on current and previous general and mental health issues as well as on demographic and psychosocial variables were collected [27]. The study was approved by the Regional Ethical Review Board in Uppsala (EPN Uppsala 2009/171). The EPDS is the most commonly accepted and utilized screening instrument for symptoms of PND [28]. It has been validated in a variety of cultural contexts and over 60 languages, including Sweden [29, 30]. The EPDS has been reported to have good internal consistency, showing Cronbach's alpha of 0.822 in a Danish study with similar population to our study [31]. The questionnaire consists of 10 individual items in which the subject is asked to self-assess relevant symptoms in terms of rate of recurrence and intensity during the last 7 days. Each item produces a score of 0–3, with a total score between 0 and 30. An elevated score is reflective of more severe symptoms. Concerning items 4, 5, and 6 in the questionnaire rating anxiety, a total score of 6 and above was defined as a high [32]. The inclusion criteria were women scoring high (12–30 points) on the EPDS at the BASIC-study follow-up at 6 weeks postpartum (*n* = 697). For women who participated twice, only the first pregnancy was included in the analyses (*n* = 24). Moreover, 11 women were excluded because of twin pregnancies and eight participants were excluded due to > 40% missing data. In total, 654 women were thus included in the analyses. In this study, an EPDS cutoff of ≥12 was used due to the national screening guidelines in Sweden, based on a Swedish validation study [29]. However, some studies show that a cutoff of ≥13 could be more specific [33]. Therefore, we conducted an additional analysis based on participants scoring 13–30 on the EPDS at 6 weeks postpartum, which is presented in Table S1.

### 2.2. Measures

Outcome was determined according to EPDS scores at 6 months postpartum; a score of 0–11 was deemed to be indicative of symptom remission while a score from 12 to 30 was interpreted as persistence of symptoms. EPDS at 6 months postpartum (outcome) were missing in 102 (15.6%) cases. Missing values were imputed multivariate imputation by chained equations (MICE). At 6 months postpartum, 342 (59.2%) women scored below 12 points and 236 (40.8%) scored ≥12. LTPDS were defined as having an EPDS score of 12–30 at both 6 weeks and 6 months postpartum. A total of 43 variables (covariates) were included in the study (Table 1). The variables were either collected as self-reports in online questionnaires within the BASIC study or were retrieved from medical records. For this substudy, a predefined protocol on variables was utilized to monitor medical records from delivery to 9 months postpartum. Data extraction from the medical records (retrospectively) was made by the first author (K.G.), a medical student, and four research assistants from 1 March 2020 through 15 December 2021. Stressful events were defined as entries in the patient records during the first 6 months postpartum, regarding death or serious illness within the family or other severe events with consequences that affect life in a significant way. Pregnancy complications were defined as late-pregnancy bleeding episodes, painful Braxton Hick's contractions, pelvic girdle pain, gestational diabetes, hypothyroidism, hypertonia, and preeclampsia. Infant issues were self-reported and included colic, rashes, breathing difficulties due to prematurity, eczema, skin boil, infection with RS virus, and jaundice. Somatic diseases were defined as any somatic disease registered in the medical record during the pregnancy or 6 months postpartum, and included asthma, migraine, inflammatory bowel disease, irritable bowel syndrome, epilepsy, skin conditions, skeletal conditions, lichen sclerosus et atrophicus, earlier gastric bypass surgery, connective tissue diseases, fibromyalgia, endometriosis, kidney diseases, repeated urinary tract infections, hypertension, hearing difficulties, immunological diseases, and heart conditions.

### 2.3. Procedures

Descriptive comparisons across groups were tested by *χ*^2^ test or Wilcoxon rank-sum test, where appropriate. A conservative approach was adapted to handle missing data. All variables (columns, corresponding to a distinct variable) with more than 40% missing were excluded (*n* = 3). The data were split into a 70% training dataset and a 30% test dataset. Missing values were imputed using multiple imputations by chained equations (MICE). MICE is a method used to handle missing data by filling in the gaps with estimated values. It does this through a repetitive process, where missing values are replaced with multiple imputations based on the observed values for an individual and their relationships to the data of other individuals [34]. Splitting was performed prior to imputation to ensure that the test dataset's missing values were imputed independently of the information from the training dataset. The training dataset was used to build and train the model. When the model was ready, it was tested on the test dataset for accuracy and an evaluation was made on how well it performed. Elastic net linear regression was used to handle collinearity among predictors with good prediction performance using regression regularization [35]. Elastic net linear regression was chosen because it combines the penalties from both the lasso and ridge techniques to regularize regression models [35]. The dependent variable was EPDS score at 6 months postpartum. Tenfold cross-validation was used to find the appropriate regularization parameter that controls the penalty strength. With this, alpha = 1 at lambda one standard error = 0.633 were chosen, resulting in eight variables used in the final model. To create a receiver operating characteristic (ROC) curve, the predicted EPDS score was categorized at different cutoffs [10, 11, 12, 13, 14, 15] and compared with the dichotomized EPDS score (0–11 vs. 12–30). Performance metrics including sensitivity, specificity, and positive and negative predictive values were computed for the different cutoffs of the predicted EPDS score. The significance level was set at *p*  < 0.05. Statistical analyses were conducted using R version 4.2.2 through RStudio [36, 37] version 2022.7.1.554 using packages glmnet, mice, caret, flux, Table 1, and ggplot2 [38, 39, 40, 41, 42].

## 3. Results

### 3.1. Characteristics in the Study Groups

Characteristics of the study population are presented in Tables 2 and 3. The Cronbach's alpha for EPDS at 6 months postpartum was 0.88 (95% CI 0.86–0.89). Descriptive statistics show that women with EPDS ≥ 12 at 6 months postpartum were to a higher extent multiparous and had lower education level and lower employment level at pregnancy week 17, compared with women who had lower EPDS at 6 months postpartum. History of depression, neuropsychiatric diagnosis, and presence of a stressful event were also more common in the group with LTPDS at 6 months postpartum, as opposed to women with lower EPDS scores. Women with LTPDS reported sleep deprivation to a higher extent in pregnancy weeks 17 and 32 and had more often high EPDS scores at pregnancy week 32 as well as high EPDS anxiety scores during pregnancy.

### 3.2. Identification of Predictive Factors

Ten variables were found using elastic net linear regression to be predictive of high EPDS score at 6 months postpartum (Table 4). University education and sleeping more than 6 hr per night during pregnancy week 32 were protective for long-term symptoms. The equation to predict EPDS score by 6 months postpartum is presented in Equation (1). One example: a woman with ADHD/ADD, EPDS score of 12–30 in pregnancy week 17, anxiety during pregnancy, and EPDS score of 12–30 in pregnancy week 32 will have a predictive EPDS score at 6 months postpartum of 10.04 (intercept) + 2.00 + 1.68 + 0.69 + 0.45 = 14.86. The subanalysis based on EPDS 13–30 at 6 weeks postpartum identified 19 predictive risk factors and 5 protective factors for LTPDS (Table S1).

### 3.3. Performance Metrics

**Figure 1:**
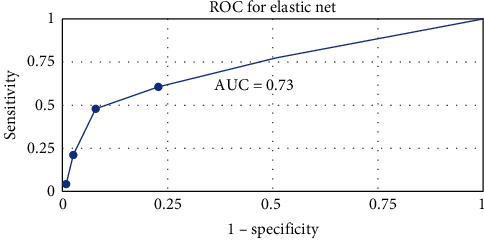
ROC curve for prediction of LTPDS using the equation created in the analysis. AUC, area under the curve; ROC curve, receiver operating characteristic curve.

The performance metrics are presented in Table 5. At 6 months postpartum, 342 (59.2%) women scored below 12 points and 236 (40.8%) scored ≥ 12. ROC curve (receiver operating characteristic curve) is a graphical representation used to show the performance of binary classification models. ROC is presented in Figure 1. The area under the curve was 0.73. The area under the curve for the subanalysis (EPDS 13–30) was 0.61, see Table S1.

Predicted EPDS at 6 months postpartum formula is given as follows (Equation (1)):

(1) $$\begin{array}{c} \frac{\begin{array}{c} \text{Predicted }EPDS\;score\;at\;6\;months\;postpartum\\ =1,004+200\;\left(if\;ADHD\;or\;ADD\right)\\ +45\;\left(if\;13-30\;EPDS\;at\;pregnancy\;week\;32\right)+69\;\left(if\;anxiety\;during\;pregnancy\right)\\ +168\;\left(if\;EPDS\;13-30\;at\;pregnancy\;week\;17\right)+42\;\left(if\;with\;pregnancy\;complications\right)\\ +41\;\left(if\;with\;crisis\;event\right)+8\;\left(if\;primi/multiparous\right)\\ +8\;\left(if\;history\;of\;depression\right)-30\;\left(if\;university\;education\right)\\ -36\;\left(if\;slept>6\;hr\;at\;pregnancy\;week\;32\right) \end{array}}{100}. \end{array}$$

## 4. Discussion

This study aimed to investigate factors that could predict the persistence of significant depressive symptoms at 6 months postpartum, among women with depressive symptoms at 6 weeks postpartum. Using this tool, a woman's risk of LTPDS can be predicted based on existing predictive factors; either the presence of one highly significant predictive factor or multiple less significant factors can indicate a high risk of developing LTPDS. The model developed and presented in this study has the potential, with appropriate further adjustments, to provide guidance for healthcare practitioners in determining the extent of additional interventions warranted for women with high EPDS scores early postpartum. This novel approach could contribute to a more cost-effective and stepped-care healthcare approach, where more intensive interventions are targeted to those with the highest risk for persistence.

Researchers have made several attempts at PND prediction models using machine learning techniques [43, 44, 45]. Mostly, they have identified factors predictive of PPD relating to previous depression and anxiety, as well as socioeconomic status, obstetric, and delivery-related variables. However, to our knowledge, the construction of a prediction model of LTPDS in women with high EPDS early postpartum has not been attempted before. Moreover, this approach is most useful and applicable in Sweden and multiple other countries where screening with EPDS in the postpartum period is embedded in clinical routines. Furthermore, the prediction metrics in the present study are acceptable, with an AUC of 0.73 and a positive predictive value of 79%–83%, depending on chosen EPDS cutoffs, making it a promising prediction tool that can be further developed. In addition, the feasibility of the approach is strengthened by the inclusion of variables that are easily accessible at the time point of the first EPDS screening (around 6 weeks postpartum), to further facilitate the identification of women with a high risk of LTPDS.

### 4.1. Identified Prediction Factors for LTPDS

In our study, 40.8% of included women continue to have depressive symptoms 6 months postpartum. This aligns well with earlier studies; a meta-analysis from 2014 showed that 30%–50% continue to have symptoms up to 12 months after delivery [20]. The predictive factors identified within this study should serve as focal points for healthcare personnel when encountering women exhibiting elevated EPDS scores in the immediate postpartum period. A few earlier studies have shown ADHD and ADD may serve as risk factors for developing PND. One study from 2021 showed that neuropsychiatric disease was associated with higher prevalence of PND, compared to the general population (57.6% vs. 19.6%) [46]. Another study found that having a diagnosis of ADHD or ADD increased the risk for both postpartum depression and anxiety [47]. Furthermore, Volkow et al. [48] reported lower levels of dopamine in patients with ADHD, compared to healthy controls, as a reason for higher sensitivity for depressive symptoms. Our results showed that ADHD and ADD were highly weighted prediction factors for LTPDS. Notably, to the best of our knowledge, no other studies have investigated ADHD and ADD specifically as predictive factors for LTPDS. The aforementioned studies may corroborate our findings and highlight the necessity for additional support during the peripartum period for this group.

We found one study, similar to the current study, conducted by Fisher et al. [24]. They identified parity, education, baseline global functioning, and depression severity as predictive factors. However, their study differed from ours in that they did not include women with moderate depression symptoms. Nonetheless, the first three variables identified by Fisher et al. [24] were also identified in our analyses and used in our tool. Unfortunately, we lacked data on baseline global functioning. Several prediction factors in our study have been shown to be linked to LTPDS also in other earlier research, such as a history of depression [18, 22, 49]. Furthermore, our results showed that high depression score during pregnancy was a predictor of LTPDS, which is well-documented as a severe risk factor for postnatal depression [50, 51, 52]. A meta-analysis from 2022 showed that antenatal depression more than doubles the risk for PPD [53]. A few studies have also demonstrated a link between depressive symptoms during pregnancy and LTPDS, aligning with our results [21, 54]. Also, sleep disturbances in the peripartum period have in earlier studies been linked to depressive symptoms during late pregnancy and postnatally, which is in line with our results [55, 56, 57, 58, 59]. Furthermore, anxiety during pregnancy has been associated with postpartum depression in general but also with LTPDS in a cohort of about 8,300 women in England in 2004 [3, 60, 61]. Pregnancy complications were found to be predictive of LTPDS in our study, which highlights the importance of following up women presenting with them. Likewise, earlier studies have demonstrated that obstetric risk factors are linked to PPD [62]. Finally, our results indicate that a high level of education and high amount of sleep serve as protective factors for LTPDS (and therefore the opposite, meaning no university education and low amount of sleep can be considered as risk factors). A high level of education as a protective factor for LTPDS aligns with findings from several earlier studies [21, 49]. Because LTPDS is a relatively understudied area and the limited scope of earlier studies, certain variables identified in our analysis have not been previously associated with LTPDS. Therefore, false positives within our model are not possible to rule out. However, considering the close association of the identified prediction factors, such as sleep disturbances, and anxiety during pregnancy, to depression and anxiety, a heightened likelihood exists for a connection between these factors and LTPDS. To increase specificity, a higher cutoff score for EPDS could be used [33]. However, despite our subanalysis with EPDS 13–30 mostly showing the same variables of importance, with ADHD/ADD as the most important factor, the prediction metrics were not as good (Table S1).

### 4.2. Trajectory Angle

As mentioned in the introduction, many studies have shown the heterogeneity of PND and identified 2–5 trajectories, typically one with chronic high depressive symptoms, one with constant low symptoms, and 1–3 groups with moderately high depression score, with decreasing or increasing symptoms [8, 63, 64]. A previous study from our research group, based on the same cohort, examined five trajectories: healthy, pregnancy depression, early postpartum onset, late postpartum onset, and chronic depression [25]. There were different risk factors associated with each. However, these trajectory studies retroactively classify women into specific groups or trajectories using a minimum of four screening time points. However, when attempting to identify a woman in the middle of a time period, such as the early postpartum period, it becomes impossible to determine her specific trajectory. Consequently, the clinical applicability of trajectory studies is somewhat constrained when determining appropriate interventions and treatments for women exhibiting elevated depressive symptoms following childbirth. Baron et al. [26] concluded in their review of 11 similar studies that there is no consistency in predicting factors for the different trajectories across studies. They, therefore, suggest that predictors could not differentiate women at risk of long-term severe symptoms from those with lower risk throughout the peripartum period, relying on trajectory-based approaches [26]. Nevertheless, studies on trajectories suggest that PPD is not uniform; instead, the disease seems to involve various subtypes, each with distinct characteristics that warrant consideration in clinical settings. Therefore, our study makes a valuable contribution to the field by adopting a different approach, paving the way for the development of a clinically relevant prediction tool.

### 4.3. Strengths and Limitations

This is the only study, to our knowledge, exploring predictive factors for LTPDS using machine learning techniques among women with depressive symptoms in the early postpartum period, rendering the study novel and unique. A further strength is the uniquely large study cohort compared to other studies in the field. Most previous studies focusing on risk factors had limited sample sizes of around 100 women [21, 22, 23]. An additional strength is the considerable number of available prospectively collected data, combining information from both the BASIC study and from medical records, increasing their validity. Also, the variables considered in the present study would be easily available in a clinical setting; the complexity of some other studies exploring predictive factors for psychiatric illnesses has limited their implementation and fit for real-world use [65]. However, a limitation of this study is that, in contrast to the general population in Sweden, our study population had to a greater extent a higher education level, were mostly born in Scandinavia, and had lower-than-average mean BMI [27], possibly limiting the generalizability of the findings, which need to be replicated in a more diverse population. While low socioeconomic status has been linked to long-term symptomatology [17, 25, 66], it has also been found to be highly predictive of study dropout [67]. Furthermore, in the BASIC study, a greater dropout rate was noted among participants with depressive symptoms at the start of the study [27]. However, the loss to follow-up was relatively limited and rates of depressive symptoms align with earlier reports when examining the entire cohort [21]. It should be noted that some of the included participants underwent interventions and/or treatments within the healthcare system, potentially influencing the course of their depressive symptoms. Therefore, the identification of certain predictors may be attributed to their association with the utilization or absence of such interventions. For instance, the inclusion of higher education as a protective factor in the tool may stem from the likelihood that women with higher education levels were more prone to accepting interventions following a positive screening, ultimately resulting in greater alleviation of their symptoms. It should also be noted that our definition of LTPDS is based on the presence of symptoms in the second postpartum month when national routine screening is taking place in the Swedish setting. For some individuals, symptoms onset might come later during the postpartum period, and those individuals are not included in the scope of this study. Therefore, the clinical tool is reflective of the real-world setting in which the study was conducted, but it cannot be generalized directly to other time periods or contexts. Furthermore, it should be acknowledged that the some of the data from BASIC were collected about 10 years ago. While the clinical screening routines remain similar, it cannot be ruled out that the passage of time may affect relevant predictors. Future studies should follow-up on these results and evaluate their reliability.

### 4.4. Clinical Implications

We propose a novel easy-to-use prediction tool categorizing women screening positive on the EPDS 6 weeks postpartum into high or low risk for LTPDS. By utilizing an easy-to-use weighted screening tool, healthcare providers would be able to identify women at higher risk for long-term symptoms and plan for a personalized intervention program and follow-up; support by educated nurses can be provided to low-risk women, whereas high-risk individuals can be referred to specialized care for further evaluation, intensive monitoring, and prompt treatment. This approach can lead to timely intervention and improved outcomes, while also optimizing the allocation of limited and costly specialist resources to those who require them most. This study also highlights the importance of antenatal depression and anxiety screening during pregnancy, to identify individuals at risk of prolonged postpartum symptomatology. Qualitative studies have shown that there is a risk of help-seeking barriers due to symptoms of depression, stigma, and difficulties overcoming healthcare system barriers. By using a structured tool to distinguish low- from high-risk women, stigma is at least somewhat addressed in women of most need [68, 69, 70]. Furthermore, the strong link between high EPDS scores during pregnancy and LTPDS observed in this study highlights the potential of utilizing screening during pregnancy as part of the risk assessment for the benefit of the mother-to-be and the offspring. The tool has to be externally validated in larger cohorts including women with different background to ensure its performance in different groups. Moreover, further studies are needed to determine which cutoff would be the most cost- and resource-effective in different settings.

## 5. Conclusion

In this study, several easily recognizable and clinically relevant variables have been associated with the prediction of LTPDS among women with depressive symptoms in the early postpartum period. An easily applicable prediction tool has been developed for early identification of women at risk, opening opportunities for accurate, and personalized intervention measures by the healthcare system. This study, with an easy-to-use predictive tool, can be the first step toward limiting the negative impact of LTPDS in women, their children, and society as a whole.

## Figures and Tables

**Table 1:** Variables included in the study, with respective units and/or grouping and source-related missing values (%), and whether included in the analysis or not (missing > 40% excluded).

Variables	Grouping and/or units	Source	Missing (%)	Included in analysis
Age at partus	≤30, 31–35, > 35 years	Basic	0	Yes
Education	University/other	Basic	10.3	Yes
Employment pregnancy week 17	Yes/no	Basic	10	Yes
Birth country	Scandinavian/other	Basic	1.1	Yes
Marital status	Married or cohabiting/no	Basic	0.2	Yes
Parity	Primi/multiparous	Basic	5	Yes
Smoking ever	Yes/no	Basic	7.6	Yes
Somatic disease	Yes/no	Medical records	0.9	Yes
ADHD/ADD	Yes/no	Medical records	0.3	Yes
History of depression	Yes/no	Basic	8.6	Yes
Anxiety disorder	Yes/no	Medical records	0.3	Yes
Levaxin treatment	Yes/no	Medical records	1.1	Yes
PMS/PMDD	Yes/no	Basic	10.7	Yes
Sleep before pregnancy	>6 hr/≤ 6 hr	Basic	10.7	Yes
Anxiety during pregnancy (EPDS)	Yes/no	Basic	7.1	Yes
BMI pregnancy week 17	kg/m^2^	Basic	11	Yes
Violence pregnancy week 17	Violence in current or previous relationship/no	Basic	10.7	Yes
EPDS pregnancy week 17	0–12/13–30	Basic	10.4	Yes
EPDS pregnancy week 32	0–12/13–30	Basic	10.1	Yes
Sleep pregnancy week 17	6 hr or more/less than 6 hr	Basic	10.3	Yes
Sleep pregnancy week 32	6 hr or more/less than 6 hr	Basic	10.1	Yes
Pregnancy complications	Yes/no	Basic	6.6	Yes
Pregnancy lenght	Days	Basic	6.3	Yes
Delivery experience	Positive/negative	Basic	16.9	Yes
Delivery fear	No fear/any fear	Basic	10.6	Yes
Delivery mode	Planned CS/emergency CS/vaginal/vacuum	Basic	0	Yes
Delivery start	Spontaneous/induction	Basic	0.2	Yes
Bleeding during delivery	<1,000 ml/≥1,000 ml	Basic	6.5	Yes
Laceration	None/grade I/grade II/grade III/grade IV	Basic	5.6	Yes
Birth weight	Grams	Basic	6.5	Yes
Gender	Male/female	Basic	6.5	Yes
Child with malformation or disease	Yes/no	Medical records	1.2	Yes
Child in neonatal ward	Yes/no	Basic	10.4	Yes
Breastfeeding PP week 6	Yes, full-time/yes, and also bottle feed/no	Basic	0.3	Yes
Infant issues	Yes/no	Basic	1.7	Yes
Alcohol intake PP week 6	Yes/no	Basic	0.9	Yes
Partner support PP week 6	Yes, much help/yes, some help/no	Basic	1.2	Yes
Support from other	Yes, much help/yes, some help/no	Basic	1.4	Yes
EPDS PP week 6	Mild/moderate/severe	Basic	0	Yes
EPDS PP Month 6	EPDS scores 0–30	Basic	16.6	Outcome
Crisis event	Yes/no	Medical records	2	Yes
Calm baby	Scores 1–7, 1—very easy, 4—average, 7—very difficult	Basic	53.6	No
Infant temper	IBQ score	Basic	54.5	No
Resilience pregnancy week 32	SOC scores 29–203	Basic	45.6	No

The variables are presented chronologically. The variables not included in the analysis are presented at the bottom of the table. Definitions of the variables are presented in the method section covariates. *Abbreviations*. PP, postpartum; CS, cesarean section; ADHD, attention deficit hyperactivity disorder; ADD, attention deficit disorder; PMS, premenstrual syndrome; PMDD, premenstrual dysphoric disorder; BMI, body mass index; SOC, sense of coherence; and IBQ, infant behavior questionnaire.

**Table 2:** Background characteristics in the total study sample as well as in the groups with and without depressive symptoms at 6 months postpartum.

	PPM6 EPDS 0–11 points (*N* = 331)	LTPDS (EPDS 12–30 points) (*N* = 221)	*p*-Value	Missing EPDS (*N* = 104)	Total (*N* = 654)
Age at partus, years					
≤30	156 (47.1%)	95 (43.4%)	0.60	47 (45.2%)	298 (45.6%)
31–35	112 (33.8%)	83 (37.9%)		30 (28.8%)	225 (34.4%)
> 35	63 (19.0%)	41 (18.7%)		27 (26.0%)	131 (20.0%)
Parity					
Nulliparous	192 (58.0%)	101 (46.1%)	0.006	48 (46.2%)	341 (52.1%)
Primi/multiparous	121 (36.6%)	106 (48.4%)		55 (52.9%)	282 (43.1%)
Missing	18 (5.4%)	12 (5.5%)		1 (1.0%)	31 (4.7%)
Birth country					
Scandinavia	297 (89.7%)	199 (90.9%)	0.85	94 (90.4%)	590 (90.2%)
Other	33 (10.0%)	20 (9.1%)		8 (7.7%)	61 (9.3%)
Missing	1 (0.3%)	0 (0%)		2 (1.9%)	3 (0.5%)
Smoking ever					
No	204 (61.6%)	123 (56.2%)	0.14	56 (53.8%)	383 (58.6%)
Yes	108 (32.6%)	87 (39.7%)		33 (31.7%)	228 (34.9%)
Missing	19 (5.7%)	9 (4.1%)		15 (14.4%)	43 (6.6%)
BMI, kg/m^2^					
Mean (SD)	24.5 (4.86)	24.7 (5.12)	0.63	24.8 (5.69)	24.6 (5.06)
Missing	30 (9.1%)	12 (5.5%)		23 (22.1%)	65 (9.9%)
Education level					
Lower	70 (21.1%)	69 (31.5%)	0.013	31 (29.8%)	170 (26.0%)
University	235 (71.0%)	138 (63.0%)		51 (49.0%)	424 (64.8%)
Missing	26 (7.9%)	12 (5.5%)		22 (21.2%)	60 (9.2%)
Employment (pregnancy week 17)					
No	286 (86.4%)	172 (78.5%)	<0.001	68 (65.4%)	526 (80.4%)
Yes	20 (6.0%)	36 (16.4%)		14 (13.5%)	70 (10.7%)
Missing	25 (7.6%)	11 (5.0%)		22 (21.2%)	58 (8.9%)
Marital status					
Married/cohabiting	324 (97.9%)	210 (95.9%)	0.18	101 (97.1%)	635 (97.1%)
Single	6 (1.8%)	9 (4.1%)		3 (2.9%)	18 (2.8%)
Missing	1 (0.3%)	0 (0%)		0 (0%)	1 (0.2%)
History of depression					
No	164 (49.5%)	79 (36.1%)	0.001	40 (38.5%)	283 (43.3%)
Yes	146 (44.1%)	129 (58.9%)		46 (44.2%)	321 (49.1%)
Missing	21 (6.3%)	11 (5.0%)		18 (17.3%)	50 (7.6%)
Neuropsychiatric diagnosis					
No	326 (98.5%)	208 (95.0%)	0.007	101 (97.1%)	635 (97.1%)
Yes	3 (0.9%)	11 (5.0%)		3 (2.9%)	17 (2.6%)
Missing	2 (0.6%)	0 (0%)		0 (0%)	2 (0.3%)
Somatic disease					
No	229 (69.2%)	141 (64.4%)	0.16	62 (59.6%)	432 (66.1%)
Yes	96 (29.0%)	78 (35.6%)		42 (40.4%)	216 (33.0%)
Missing	6 (1.8%)	0 (0%)		0 (0%)	6 (0.9%)
Anxiety EPDS score during pregnancy					
<6	234 (70.7%)	110 (50.2%)	<0.001	54 (51.9%)	398 (60.9%)
≥6	79 (23.9%)	104 (47.5%)		32 (30.8%)	215 (32.9%)
Missing	18 (5.4%)	5 (2.3%)		18 (17.3%)	41 (6.3%)
Stressful event					
No	294 (88.8%)	185 (84.5%)	0.02	89 (85.6%)	568 (86.9%)
Yes	27 (8.2%)	33 (15.1%)		14 (13.5%)	74 (11.3%)
Missing	10 (3.0%)	1 (0.5%)		1 (1.0%)	12 (1.8%)

*Abbreviations*. PPM, postpartum month 6; EPDS, Edinburgh Postnatal Depression Scale; PP, postpartum; BMI, body mass index; SD, standard deviation; and LTPDS, long-term postpartum depressive symptoms.

**Table 3:** Pregnancy, delivery, and postpartum variables in the total sample as well as in the groups with and without depressive symptoms at 6 months postpartum.

	PPM6 EPDS 0–11 points (*N* = 331)	LTPDS (EPDS 12–30 points) (*N* = 221)	*p*-Value	Missing EPDS (*N* = 104)	Total (*N* = 654)
EPDS (score, pregnancy week 17)					
0–12	257 (77.6%)	123 (56.2%)	<0.001	48 (46.2%)	428 (65.4%)
13–30	47 (14.2%)	84 (38.4%)		34 (32.7%)	165 (25.2%)
Missing	27 (8.2%)	12 (5.5%)		22 (21.2%)	61 (9.3%)
EPDS (score, pregnancy week 32)					
0–12	230 (69.5%)	108 (49.3%)	<0.001	49 (47.1%)	387 (59.2%)
13–30	79 (23.9%)	100 (45.7%)		26 (25.0%)	205 (31.3%)
Missing	22 (6.6%)	11 (5.0%)		29 (27.9%)	62 (9.5%)
EPDS postpartum week 6					
Moderate (12–18)	290 (87.6%)	186 (84.9%)	0.44	91 (87.5%)	567 (86.7%)
Severe (19–30)	41 (12.4%)	33 (15.1%)		13 (12.5%)	87 (13.3%)
Gestational age					
Preterm	16 (4.8%)	12 (5.5%)	0.90	5 (4.8%)	33 (5.0%)
Not preterm	293 (88.5%)	193 (88.1%)		96 (92.3%)	582 (89.0%)
Missing	22 (6.6%)	14 (6.4%)		3 (2.9%)	39 (6.0%)
Sleep (hours, pregnancy week 17)					
<6	281 (84.9%)	174 (79.5%)	0.007	70 (67.3%)	525 (80.3%)
≥6	24 (7.3%)	33 (15.1%)		12 (11.5%)	69 (10.6%)
Missing	26 (7.9%)	12 (5.5%)		22 (21.2%)	60 (9.2%)
Sleep (hours, pregnancy week 32)					
<6	40 (12.1%)	51 (23.3%)	0.001	18 (17.3%)	109 (16.7%)
≥6	269 (81.3%)	157 (71.7%)		58 (55.8%)	484 (74.0%)
Missing	22 (6.6%)	11 (5.0%)		28 (26.9%)	61 (9.3%)
Delivery mode					
Vaginal delivery	239 (72.2%)	152 (69.4%)	0.63	72 (69.2%)	463 (70.8%)
Vacuum extraction	28 (8.5%)	26 (11.9%)		4 (3.8%)	58 (8.9%)
Planned CS	26 (7.9%)	17 (7.8%)		15 (14.4%)	58 (8.9%)
Emergency CS	38 (11.5%)	24 (11.0%)		13 (12.5%)	75 (11.5%)
Partner support postpartum week 6					
Yes, much help	185 (55.9%)	104 (47.5%)	0.141	36 (34.6%)	325 (49.7%)
Yes, some help	127 (38.4%)	94 (42.9%)		60 (57.7%)	281 (43.0%)
No	17 (5.1%)	17 (7.8%)		6 (5.8%)	40 (6.1%)
Missing	2 (0.6%)	4 (1.8%)		2 (1.9%)	8 (1.2%)

*Abbreviations*. PPM, postpartum month 6; EPDS, Edinburgh Postnatal Depression Scale; BMI, body mass index; CS, caesarean section; SD, standard deviation; and LTPDS, long-term postpartum depressive symptoms. Comparisons across groups were tested by *χ*^2^ test or Wilcoxon rank-sum test, where appropriate.

**Table 4:** Prediction factors with coefficients from the elastic net analysis to predict continuous EPDS at 6 months postpartum.

Predictive variable	Coefficient
(Intercept)	10.04
ADHD/ADD	2.00
EPDS 12–30 pregnancy week 17	1.68
Anxiety during pregnancy	0.69
EPDS 12–30 pregnancy week 32	0.45
Pregnancy complications	0.42
Stressful event	0.41
History of depression	0.08
Parity	0.08
University education	−0.30
Sleep > 6 hr during pregnancy week 32	−0.36

**Table 5:** Performance metrics of the model across different predicted EPDS cutoffs at 6 months postpartum.

Cutoff predicted EPDS at 6MPP	Sensitivity (%)	Specificity (%)	PPV (%)	NPV (%)
0*–*9.9/10*–*30	77	49	49	78
0*–*10.9/11*–*30	61	77	62	76
0*–*11.9/12*–*30	48	92	79	74
0*–*12.9/13*–*30	21	97	83	66
0*–*13.9/14*–*30	4	99	75	62

Performance metrics of the model for predicting dichotomous outcome of EPDS 12–30 vs. 0–11 at 6 months postpartum. EPDS, Edinburgh Postnatal Depression Scale; 6MPP, 6 months postpartum; PPV, positive predictive value; and NPV, negative predictive value.

## Data Availability

The data that support the findings of this study are available upon reasonable request from the corresponding author (Karin Gidén). The data are not publicly available due to their containing information that could compromise the privacy of research participants. The code is available at https://github.com/frugiden/LTPDS.git.

## Supplementary Materials

Supplementary Materials In Table S1, an additional analysis was made. This analysis identified 19 predictive risk factors and five protective factors for LTPDS (see Table S1). Most of the identified variables are the same as in the main analysis of this study (EPDS 12–30), with the most highly valued factor being ADHD/ADD. Other factors identified included laceration grade IV, social factors such as unemployment and partner help, age, psychiatric problems such as PMD/PMDD, fear of childbirth, and problems with the baby (disease or admission to NICU). The area under the curve was 0.61.
