# Supplementary material for: Remission or Persistence? A Prediction Tool to Identify Women at Risk for Long-Term Depressive Symptoms Postpartum
Source: Depress Anxiety. 2024 Aug 30;2024:7734542. doi: 10.1155/2024/7734542 (PMC11918868; doi:10.1155/2024/7734542)
Supplement: Supplementary Materials — In Table S1, an additional analysis was made. This analysis identified 19 predictive risk factors and five protective factors for LTPDS (see Table S1). Most of the identified variables are the same as in the main analysis of this study (EPDS 12–30), with the most highly valued factor being ADHD/ADD. Other factors identified included laceration grade IV, social factors such as unemployment and partner help, age, psychiatric problems such as PMD/PMDD, fear of childbirth, and problems with the baby (disease or admission to NICU). The area under the curve was 0.61. [file 7734542.f1.docx]

Supplementary 1

This analysis identified 19 predictive risk factors and 5 protective factors for LTPDS (see Supplementary Table 1). Most of the identified variables are the same as in the main analysis of this study (EPDS 12-30), with the most highly valued factor being ADHD/ADD. Other factors identified included laceration grade IV, social factors such as unemployment and partner help, age, psychiatric problems such as PMD/PMDD, fear of childbirth, and problems with the baby (disease or admission to NICU). The area under the curve was 0.61.

**Supplementary Table 1. Prediction model with participants with EPDS 13-30 at six weeks postpartum (n=519)**

| Intercept | 10.4 |
| --- | --- |
| ADHD | 1.35 |
| Laceration grade IV | 0.82 |
| Anxiety during pregnancy | 0.64 |
| EPDS 12-30 during pregnancy week 32 | 0.52 |
| EPDS 12-30 during pregnancy week 17 | 0.47 |
| Unemployment | 0.22 |
| PMS/PMDD | 0.2 |
| No partner help | 0.18 |
| Anxiety | 0.17 |
| < 6 h sleep/night pregnancy week 17 | 0.16 |
| Child admitted to NICU | 0.15 |
| Age 31-35 | 0.12 |
| Child gender boy | 0.12 |
| History of depression | 0.09 |
| Fear of childbirth | 0.08 |
| Crisis event | 0.04 |
| Child with disease or malformation | 0.04 |
| Multiparity | 0.04 |
| EPDS >18 at postpartum week 6 | 0.04 |
| Age >35 | -0.03 |
| Born outside of Sweden | -0.11 |
| Negative delivery experience | -0.19 |
| University education | -0.26 |
| > 6 h sleep/night pregnancy week 32 | -0.55 |
